# Impact of Tumor Necrosis Factor Receptor 1 (*TNFR1*) Polymorphism on Dry Eye Disease

**DOI:** 10.3390/biom13020262

**Published:** 2023-01-31

**Authors:** Kelly Acuna, Anjalee Choudhary, Elyana Locatelli, Daniel A. Rodriguez, Eden R. Martin, Roy C. Levitt, Anat Galor

**Affiliations:** 1 Department of Ophthalmology, Miami VA Medical Center, Miami, FL 33136, USA; 2 Department of Ophthalmology, Bascom Palmer Eye Institute, University of Miami, Miami, FL 33136, USA; 3 John T. MacDonald Foundation Department of Human Genetics, University of Miami Miller School of Medicine, Miami, FL 33136, USA; 4 John P. Hussman Institute for Human Genomics, University of Miami Miller School of Medicine, Miami, FL 33136, USA; 5 Department of Anesthesiology, Perioperative Medicine and Pain Management, University of Miami Miller School of Medicine, Miami, FL 33136, USA

**Keywords:** *TNFR1*, genetic polymorphism, anti-inflammatory treatment, dry eye, rs1800693, single nucleotide polymorphism

## Abstract

The goal of the study was to examine whether a genetic polymorphism in tumor necrosis factor receptor 1 (*TNFR1*) gene impacted the dry eye disease (DED) phenotype and response to anti-inflammatory therapy. The prospective study included 328 individuals with various dry eye (DE) symptoms and signs recruited from the Miami Veterans Hospital eye clinic between October 2013 and October 2017. The population underwent genetic profiling for a polymorphism within the *TNFR1* gene (rs1800693 [TT, TC, CC]). The study examined the genotype distribution and relationships between the genotype, phenotype, and response to anti-inflammatory therapy. The mean age of the population was 61.7 ± 9.8 years. Here, 92% self-identified as male, 44% as White, and 21% as Hispanic; 13% (*n* = 42) of individuals had a CC genotype. DED symptoms and signs were similar across the three genotype groups. Thirty individuals (four with CC) were subsequently treated with an anti-inflammatory agent. There was a non-significant trend for individuals with CC genotype to have a partial or complete symptomatic response to treatment compared with the other two groups (100% for CC vs. 40% for TT and 36.4% for TC, *p* = 0.22). In conclusion, the presence of homozygosity of minor allele C (CC genotype) in a single nucleotide polymorphism (SNP) within *TNFR1* was noted in a minority of individuals with various aspects of DED, but did not impact the DED phenotype. Our findings suggest that the current phenotyping strategies for DED are insufficient to identify underlying disease contributors, including potential genetic contributors.

## 1. Introduction

Dry eye disease (DED) is a complex, multifactorial disease with numerous symptoms (e.g., pain symptoms described as dryness, and burning, tenderness, and visual symptoms described as unstable, fluctuating, poor quality vision) and signs (e.g., decreased tear production, tear instability, corneal epithelial cell irregularity, and ocular surface inflammation) [1] Population-based studies in various countries, including the United States [2,3], Taiwan [4], and Indonesia [5], have found that DED symptoms are frequently encountered in the general population. The physical and mental impact of DED can affect a patient’s quality of life [6]. Because of the frequency and multifactorial nature of DED, a comprehensive understanding of an individual’s contributors to disease is vital for delivering precision medicine. 

Focus has been given to both environmental and genetic contributors in DED. In the case of the environment, factors such as air pollution and low humidity have been found to contribute to a DED diagnosis [7]. In the case of genetics, DED was shown to be heritable in one female twin study in the United Kingdom, with an estimated heritability of approximately 30% for symptomatic DED, as defined by the question “For the past three months or longer, have you had dry eyes?” [8] Furthermore, DED often manifests as part of Sjögren’s syndrome (SS), a disease with known genetic contributors [9]. Less well examined has been the impact of genetics on DED outside the purview of SS, as well as the treatment response. 

Inflammation has been described as a core mechanism in DED, with TNF and other inflammatory mediators implicated in various aspects of the disease [10]. Genetic polymorphisms in the genes involved in inflammatory pathways may be one link between genetic risk and DED. Of interest to the current study is the single nucleotide polymorphism (SNP) rs1800693 (chr12, alleles: T/C), which occurs in intron 6 of the *TNFR1* gene. The polymorphism is within a splice acceptor site, resulting in the loss of exon 6 and the production of a truncated soluble form of TNFR1, referred to as soluble TNFR1 (sTNFR1) [11]. sTNFR1 levels have been found to be increased in both ocular and systemic inflammatory diseases [12,13]. With respect to DED, one study examined the impact of rs1800693 on treatment response in a subset of subjects treated with OC-02 (a topical anti-TNFα antibody fragment) as part of a prospective, randomized study. In individuals with physician diagnosed DED and a global ocular discomfort score ≥50, those homozygous for the C allele (*n* = 4) had a better symptomatic response to OC-02 compared with the other two groups (TC *n* = 25; TT *n* = 14) (mean change in global ocular discomfort score CC: −29.48 ± 6.52; TC: −3.90 ± 3.51; TT: −0.09 ± 3.52; *p* < 0.0001) [14]. These results suggest that homozygosity of the minor allele in rs1800693 may influence treatment response to an anti-inflammatory agent. The purpose of the current study was to examine the frequency of the minor allele in rs1800693 in a novel population (South Florida veterans) and to examine how the presence of each genotype impacted the DED phenotype or response to anti-inflammatory treatment. 

## 2. Methods

### 2.1. Study Population

Patients with normal eyelid, conjunctival, and corneal anatomy were prospectively recruited from the Miami Veterans Affairs Medical Center eye clinic between October 2013 and October 2017 and underwent a complete ocular surface examination. Patients were excluded from participation if they had ocular or systemic co-morbidities that could confound DED (e.g., contact lens wear, history of refractive, glaucoma, or retina surgery, use of ocular medications apart from artificial tears, an auto-immune disease linked to DED such as SS, sarcoidosis, or graft-versus-host disease (GVHD)). The Miami VA Institution Review Board approval allowed for the prospective evaluation of patients. The study was conducted in accordance with the principles of the Declaration of Helsinki. 

### 2.2. Data Collected

Individuals filled out questionnaires regarding demographics (age, gender, and race (White, Black, and Other), ethnicity (Hispanic and non-Hispanic)), co-morbid medical conditions, medications, and medical device usage.

### 2.3. Ocular Surface Symptoms

Individuals filled out several standardized questionnaires regarding symptoms, including the five-item Dry Eye Questionnaire (DEQ-5) [15] (range 0–22) and Ocular Surface Disease Index (OSDI) [16]. (range 0–100). The subjects also filled out ocular surface pain-specific questionnaires, including a numerical rating scale (NRS) (“How would you describe the overall intensity of your ocular pain on average during the past 1 week?” range 0–10) and the Neuropathic Pain Symptom Inventory modified for the eye (NPSI-Eye) [17]. (range 0–40), encompassing questions regarding burning, and evoked pain to wind, light, and temperature change.

### 2.4. Assessment of Corneal Sensitivity

Mechanical detection and pain thresholds of the central cornea were assessed with a modified Belmonte non-contact esthesiometer, which was developed based on the original Belmonte instrument [18]. The tip of the aesthesiometer (0.5 mm in diameter) was placed perpendicular to, and 4 mm from, the surface of the cornea of the right eye. The stimulation consisted of pulses of air at room temperature (approximately 23 to 26 °C) [19] applied to the corneal surface. The method of limits, using ascending series only, was used to measure the threshold in the right eye. 

For corneal sensation detection, subjects were presented with a stimulus immediately following a blink and asked to indicate whether they felt the stimulus. The initial flow rate was set at a level below threshold (50 mL/min for most individuals) and increased by 10 mL/min (with 15 s intervals between stimuli) until the subject stated that they felt the stimulus, or the maximum allowable flow rate (400 mL/min) was reached. Two ascending series were conducted, and the detection threshold was defined as the arithmetic mean of the two values.

### 2.5. Ocular Surface Examination

All individuals underwent a tear film assessment, including the measurement of (1) matrix metallopeptidase 9 (MMP-9) (Inflammadry, Quidel, San Diego, CA, USA), qualitatively graded on a scale of 0–3 based on the intensity of the pink stripe (1, none; 2, faint pink; 3, fuchsia); (2) assessment of upper and lower eyelid laxity determined by the snap back test (scale 0–2); (3) anterior blepharitis graded on a scale of 0–3 (0, none; 1, mild; 2, moderate; 3, severe); (4) eyelid vascularity graded on a scale of 0–3 (0, none; 1, mild engorgement; 2, moderate engorgement; 3, severe engorgement); (5) inferior meibomian gland plugging graded on a scale 0–3 (0, none; 1, less than 1/3 lid involvement; 2, between 1/3 and 2/3 lid involvement; 3, greater than 2/3 lid involvement); (6) lower eyelid Meibomian gland [1] dropout graded via the Meiboscale, 0–4; (7) papillary changes graded on a scale of 0–2 (0, none; 1, mild; 2, severe); (8) conjunctivochalasis graded as absent or present in each area of the lower eyelid (temporal, central, and nasal) based on the obliteration of the tear film by conjunctivae in the region of interest, scale 0–3; (9) tear stability measured via tear breakup time (TBUT) (5 µL fluorescein placed, three measurements taken in each eye and averaged), lower times indicate more tear instability; (10) corneal epithelial cell disruption measured via corneal staining (National Eye Institute (NEI) scale, five areas of cornea assessed (score 0–4 in each, and total 15)), higher levels indicate more disruption; (11) anesthetized Schirmer’s, mm wetting at 5 min, lower levels indicate decreased tear production; and (12) meibum quality assessed on a scale of 0 to 4 (0, clear; 1, cloudy; 2, granular; 3, toothpaste; 4, no meibum extracted).

### 2.6. Dry Eye Categories

Based on the questionnaire and examination findings, individuals were further grouped into DED categories. Individuals were defined as having evaporative DED [2] if they had a DEQ ≥ 6, Schirmer > 5 mm in both eyes, and TBUT ≤ 5 s in at least one eye. Individuals were defined as having aqueous tear deficiency (ATD) if they had a DEQ ≥ 6 and a Schirmer ≤ 5 mm in at least one eye.

### 2.7. Genotyping and Genetic Association Analysis

Genomic DNA was purified from the whole blood using Puregene chemistry on the Qiagen Autopure LS according to standard automated Qiagen protocols (Valencia, CA, USA). As part of a larger study, the samples were genotyped using Illumina’s Infinium^®^ Expanded Multi-Ethnic Genotyping Array (MEGA^EX^) (Illumina, Inc., San, Diego, CA, USA), which interrogates approximately 2 million markers. The samples were processed according to Illumina Procedures for processing of the Infinium LCG ^®^ Assay (Illumina, Inc., San Diego, CA, USA). Data were extracted by the Illumina ^®^ Genome Studio software (GenomeStudio, v2.0.5) from data files created by the Illumina iscan. Samples with call rates below 98% were excluded from the analysis and a GenCall cutoff score of 0.15 was used for all of the Infinium II ^®^ products. We then extracted the genotype calls for rs1800693, our biallelic SNP of interest. Participants were characterized as homozygous for the major allele T, and heterozygous or homozygous for the minor allele C.

### 2.8. Response to Anti-Inflammatory Therapy

Thirty individuals were prescribed a topical anti-inflammatory therapy, either cyclosporine 0.05% (Restasis, Allergan Inc., Irvine, CA, USA) or lifitegrast 5% (Xiidra, Novartis Pharmaceuticals Corp., East Hanover, NJ, USA) as part of the clinical care. Overall, this group had higher DED symptom severity (DEQ5: 13.97 ± 4.17 vs. 10.94 ± 5.20, *p =* 0.002) and lower tear production (Schirmer: 10.28 ± 7.16 vs. 13.28 ± 7.33, *p =* 0.04) compared to individuals not treated with an anti-inflammatory drop. However, the decision to treat was based on the preference of the treating physician. In all patients, a topical corticosteroid was prescribed twice daily for the first month (fluorometholone ophthalmic suspension, 0.1%). The DED symptom response was subjectively assessed approximately 3 months later. Individuals were sorted into two groups: individuals who characterized their ocular surface symptoms as partially or completely resolved upon starting anti-inflammatory therapy, and those with stable or worsening symptoms.

### 2.9. Statistical Analysis

Statistical analysis was performed using the SPSS 28.0 (IBM Corp, Armonk, NY, USA) statistical package. Descriptive statistics were used to summarize the patient demographic and clinical information. Chi square test or Fischer’s exact test were used, as appropriate, for the categorical variables. Analysis of variance was used for the continuous variables. The more severe value from each eye was used when examining the DED signs. Given the preliminary nature of the study, we opted to report all of the examined variables, with accompanying confidence intervals, and did not adjust *p*-values for multiple comparisons, given the potential limitations with this approach [20].

## 3. Results

### 3.1. Study Population

In total, 328 subjects were included in the analysis. The characteristics from the study population are shown in Table 1. The mean age of all of the subjects was 61.7 ± 9.8 years; 91.8% (*n* = 301) self-identified as male, 55.8% (*n* = 183) as Black, 43.9% (*n* = 144) as White, and 21.3% (*n* = 70) as Hispanic. Here, 16.2% (*n* = 53) individuals met the criteria for EDE and 20.4% (*n* = 67) for ATD. 

### 3.2. Frequency of rs1800693 Polymorphisms in the Study Population

**Figure 1:**
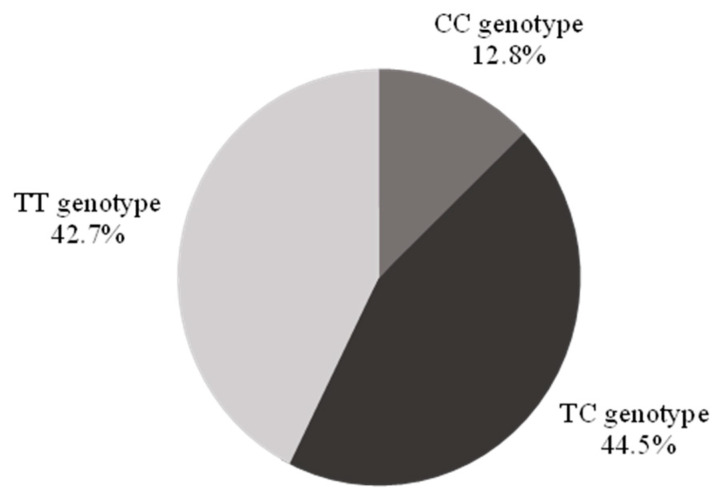
Demographics of the study population by genotype. There were no significant differences in sex, race, and ethnicity across genotypes (Table 1). Individuals with a CC genotype were older than their counterparts, less likely to use statins, and more likely to use sildenafil.

The CC genotype was identified in 12.8% (*n* = 42, 95% confidence interval (CI) 9.2–16.4%) of the population, the TT genotype in 42.7% (*n* = 140), and the TC genotype in 44.5% (*n* = 146) (Figure 1). 

### 3.3. Relationship of SNP rs1800693 to DED Symptoms and Signs

DED symptoms and signs from the study population are shown in Table 2. When looking at the population as a whole, the presence of the CC genotype (as compared to TT and TC) did not influence the DED phenotype with similar DED symptoms and signs across the groups (Table 2). The only metric that was significantly different across groups was corneal sensitivity, which was lower in individuals with a CC genotype than the other two groups (represented by a higher mechanical detection threshold). 

Symptoms and signs were also examined by genotype within race and ethnicity groups. Of all analyses, the only significant finding was a higher corneal staining in Whites with the CC versus the CT or TT genotypes (CC: *n* = 17, 4.18 ± 3.19; CT: *n* = 71, 2.34 ± 2.65; TT: *n* = 55, 1.89 ± 2.26; *p =* 0.007). While not statistically significant, corneal thresholds were lower (representing higher corneal sensitivity) in Hispanics with a CC genotype (CC: *n* = 5, 48.00 ± 13.04 mL/min; CT: *n* = 32, 86.09 ± 40.56; TT: *n* = 32, 92.97 ± 41.93 mL/min; *p =* 0.07). 

The analysis was repeated in the sub-set of individuals (*n* = 88) with DED symptoms (Dry Eye Questionnaire 5 score ≥ 6) and significant corneal staining (National Eye Institute Scale score ≥ 3). In this population, 15% (*n* = 13) of individuals had a CC genotype but again, this genotype did not influence the DED phenotype. 

### 3.4. Response to Anti-Inflammatory Therapy

**Figure 2:**
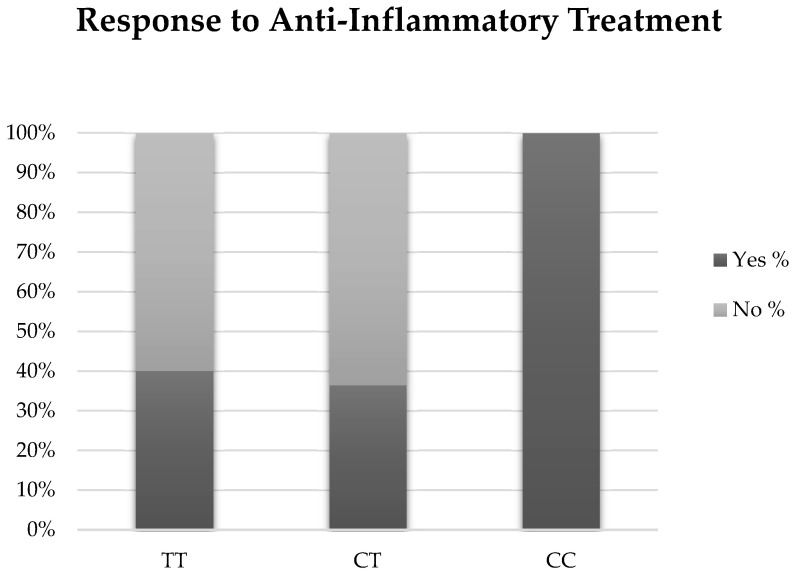
Results of anti-inflammatory therapy for each genotype.

Thirty individuals (3 with EDE, 10 with ATD, and 4 with a CC genotype) were treated with an anti-inflammatory agent, either cyclosporine 0.05% (Restasis, Allergan Inc., Irvine, CA, USA) or lifitegrast 5% (Xiidra, Novartis Pharmaceuticals Corp., East Hanover, NJ, USA) (Table 3). There was a trend for individuals with the CC genotype to have a partial or complete symptomatic response to treatment compared with individuals with TC or TT genotypes. Although not significant, 100% of individuals with the CC genotype reported improved or resolved symptoms (*n* = 4) compared with 40% of TT (*n* = 6) and 36.4% of TC (*n* = 4) (*p =* 0.22). (Figure 2). When examined by DED sub-group, two individuals with EDE (66.7%) and four individuals with ATD (40%) were considered responders (*p =* 0.42). Our findings were unchanged when examined with a multivariable forward stepwise logistic regression analysis that included genotype, age, and statin use.

## 4. Discussion

In this prospective study, we examined whether a genetic polymorphism in the *TNFR1* gene (rs1800693) was associated with the DED phenotype (symptoms and signs), as well as the response to anti-inflammatory therapy in 328 individuals. The CC genotype in SNP rs1800693 was the least common variant, found in only 12.8% of the population. The CC genotype did not influence DED symptom severity or ocular surface signs, except for corneal sensitivity, which was lower in the CC group compared with the other two groups. Subsequently, a sub-population of individuals were treated with an anti-inflammatory agent as part of their clinical care. Although non-significant, there was a trend for those with a CC genotype to have a better symptomatic response to treatment compared with the TC and TT genotypes. Our findings are similar to those of the OCS-02 trial, where individuals with a CC genotype had a greater improvement in the global ocular discomfort score after treatment with a topical anti-TNFα antibody fragment compared with the TC and TT genotypes. 

TNF-α is a pro-inflammatory cytokine that has been implicated in the pathophysiology of DED [10]. In a study of 18 individuals with DED (defined by symptoms >3 months, TBUT < 5 s, Schirmer < 10 mm, low tear clearance rate (<8 times), and positive fluorescein or Rose Bengal vital staining (≥3) OR a SS diagnosis), the levels of TNF-α were higher in the cases compared with 14 controls (3.68 ± 3.45 pg/mL versus < 0.5 pg/mL, *p* < 0.01) [21]. Anti-inflammatories, such as cyclosporine and lifitegrast, as used in this study, modulate various aspects of ocular surface inflammation, including cellular (T cells) and soluble (TNF-α) mediators. In a mice model, experimental DED was induced by subcutaneous injection of scopolamine hydrobromide four times a day and exposure to an air draft and 30% ambient humidity. The mice were divided into different topical treatment groups, including a 0.05% cyclosporine arm. The cyclosporine-treated group had significantly lower levels of conjunctival TNF-α compared to untreated mice at day 10 (~6 pg/mL vs. ~11 pg/mL, *p* < 0.001) [22]. Lifitegrast, an LFA-1 antagonist, has also been shown to impact TNF-α levels [23]. In an in vitro study, lifitegrast inhibited the release of TNF-α from human peripheral blood mononuclear cells stimulated with staphylococcal enterotoxin B. [24]. Thus, the beneficial effects of cyclosporine and lifitegrast in DED may partially be through modulation of TNF-α levels.

As TNF-α has been shown to be a target in DED, our study aimed to examine how a polymorphism in the *TNFR1* gene could impact DED phenotypes. TNFR1 is one of the primary receptors that facilitates the pro-inflammatory response to TNF-α, and it is encoded by the gene *TNFRSF1A* (tumor necrosis receptor superfamily member 1A) [25]. In this study, we looked at rs1800693, a SNP within *TNFR1*. SNP rs1800693 (alleles: T/C) is located in intron 6 within a splice acceptor site, resulting in the loss of exon 6 and the production of a shortened variant. This alteration gives rise to a truncated soluble form of TNFR1, referred to as Soluble TNFR1 (sTNFR1) [11]. sTNFR1 levels have been shown to be increased in both ocular and systemic inflammatory diseases. For example, one study that collected aqueous humor and vitreous samples from individuals with active and inactive uveitis and the controls showed that intraocular levels of sTNFR1 were significantly increased in uveitis, particularly in individuals with active uveitis [12]. In the aqueous humor, sTNFR1 levels in individuals with active uveitis (*n* = 12) were significantly higher than in the controls (*n* = 17) (1259 ± 1017 pg/mL vs. 35 ± 38 pg/mL, *p* < 0.0001). Similar findings have been noted in DED. In one study, tear sTNFR1 levels were increased in individuals with SS and GVHD compared with the controls (SS: 1.92 ± 0.90, ng/mL, *n* = 12; GVHD, 8.24 ± 3.81 ng/mL, *n* = 4; controls, 0.34 ± 0.25 ng/mL, *n* = 10, *p* < 0.05 for both) [13]. Together, these studies suggest an increase in sTNFR1 in inflammatory states, which may be a compensatory response as circulating sTNFR1 is capable of neutralizing endogenous TNF-α. In one study, the plasma samples from five volunteers administered endotoxin were incubated with a recombinant sTNFR-1. The addition of recombinant sTNFR1 reduced the endogenous TNF-α activity by 66% due to the formation of a TNF-α-sTNFR-1 complex that exhibited a reduced cytotoxic activity (determined by ELISA) [26]. These results suggest an anti-inflammatory role of sTNFR-1 via the neutralization of TNF-α. Despite the biologic plausibility that individuals with a CC genotype would have less ocular surface inflammation compared with the TT and TC genotypes, no differences in ocular surface signs, including the qualitatively assessed MMP-9, were noted between our groups. It is also not clear whether this variant impacts the effect of anti-inflammatory agents (both TNF-α neutralizing and T cell modulatory) on DED symptoms, but this is suggested by our findings and prior data. Further studies are needed to understand how the CC genotype specifically impacts sTNFR-1 levels on the ocular surface and, subsequently, ocular surface inflammation and response to therapy in various disease sub-types (SS associated vs. isolated DED).

As with all studies, limitations should be considered when interpreting the results. First, our study population consisted of South Florida veterans, and thus our findings may not be generalizable to other populations. However, it is encouraging that our frequencies and results mirror that of the OC-02 population, which was a predominantly White, female cohort. Second, our population excluded individuals with SS and GVHD, to avoid systemic co-morbidities that could confound the results of our larger study, whose goal is to examine genetic contributors to ocular pain. As such, we cannot comment on the impact of the CC genotype in individuals with co-morbid auto-immune diseases. Third, our findings are limited by the DED testing performed, and it is possible that other facets of disease, such as nerve morphology detected with in vitro confocal microscopy, may have had differences between the groups. Fourth, the findings’ weakest yet most interesting aspect is the relationship between the CC genotype and the treatment effect. However, only a minority of patients were treated with anti-inflammatory therapy, at the discretion of the treating physician, and only symptomatic improvements were recorded. Given the low numbers of patients treated with an anti-inflammatory therapy, there was not enough power to further examine the treatment response by DED subtype (EDE vs. ATD) or medication (cyclosporine 0.05% vs. lifitegrast 5%).

Despite the limitations, the novelty of our work comes from its examination of how a particular genotype impacts the phenotype and response to therapy in dry eyes. The study highlights how genetic polymorphisms may not be detected by current phenotypic strategies, but may potentially influence the treatment response to anti-inflammatory therapy. This finding is important as understanding an individual’s contributors to DED symptoms and signs, including genetic ones, is needed in order to deliver precision medicine in DED. Future studies with more robust methodologies are needed to validate our findings and to explore other genetic polymorphisms, as well as their relationship to DED phenotypes and the response to therapy in novel populations.

## Figures and Tables

**Table 1:** Demographics of the study population by genotype.

	Genotype			
	TT (*n* = 140)	TC (*n* = 146)	CC (*n* = 42)	*p*-Value
Demographics				
Age (mean± standard deviation (SD)), years	60.8 ± 9.4	61.4 ± 9.9	65.3 ± 9.8	**0.01**
Sex, male %, (*n*)	92.1% (129)	91.1% (133)	92.9% (39)	0.91
White, Non-Hispanic % (*n*)	34.6% (27)	50.0% (39)	15.4% (12)	0.11
Black, Non-Hispanic % (*n*)	45.3% (81)	40.8% (73)	14.0% (25)	
White, Hispanic % (*n*)	42.4% (28)	50.0% (33)	7.6% (5)	
Black, Hispanic % (*n*)	100.0% (4)	0% (0)	0% (0)	
Comorbidities, % (*n*)				
Smoking (current)	40.0% (56)	38.4% (56)	42.9% (18)	0.98
Hypertension	67.9% (95)	71.2% (104)	69.0% (29)	0.82
Hypercholesterolemia	58.6% (82)	56.8% (83)	50.0% (21)	0.62
PTSD	17.9% (25)	19.9% (29)	33.3% (14)	0.09
Depression	65.0% (91)	56.8% (83)	52.4% (22)	0.22
Arthritis	51.8% (72)	46.6% (68)	43.9% (18)	0.56
Sleep Apnea	23.6% (33)	21.2% (31)	23.8% (10)	0.88
BPH	16.4% (23)	17.1% (25)	19.0% (8)	0.93
Rosacea	3.6% (5)	1.4% (2)	4.8% (2)	0.36
Hepatitis C	11.4% (16)	11.0% (16)	2.4% (1)	0.21
Devices and Medications, % (n)				
CPAP	7.1% (10)	4.1% (6)	4.8% (2)	0.52
NSAIDs	36.4% (51)	31.5% (46)	35.7% (15)	0.66
ASA	44.3% (62)	41.8% (61)	38.1% (16)	0.76
Fish Oil	10.7% (15)	10.3% (15)	9.5% (4)	0.97
Multivitamins	52.1% (73)	45.9% (67)	59.5% (25)	0.25
Beta Blockers	17.9% (25)	17.1% (25)	19.0% (8)	0.96
Statins	54.3% (76)	47.9% (70)	31.0% (13)	**0.03**
Antidepressants	46.4% (65)	48.6% (71)	45.2% (19)	0.90
Anxiolytics	47.9% (67)	45.9% (67)	50.0% (21)	0.88
Analgesics	64.0% (89)	61.6% (90)	66.7% (28)	0.82
Antihistamines	22.1% (31)	21.2% (31)	9.5% (4)	0.18
Sildenafil	30.7% (43)	25.3% (37)	50.0% (21)	**0.01**

PTSD: post-traumatic stress disorder; BPH: benign prostatic hyperplasia; TBI: traumatic brain injury; CPAP: continuous positive pressure airway; NSAID: non-steroidal anti-inflammatory drugs; ASA: acetylsalicylic acid.

**Table 2:** Dry eye symptoms and signs in the study population by genotype.

	Genotype			
	TT (*n* = 140)	TC (*n* = 146)	CC (*n* = 42)	*p*-Value
DED and ocular pain symptoms quantified by questionnaires (mean ± SD)				
DEQ-5 (range 0–22)	10.99 ± 5.35	11.45 ± 5.11	11.19 ± 4.98	0.76
OSDI (range 0–100)	35.96 ± 24.99	34.94 ± 23.89	32.69 ± 24.18	0.75
NRS (range 0–10)	3.22 ± 2.63	3.21 ± 2.65	2.88 ± 2.61	0.75
NPSI-Eye (range 0–40)	21.40 ± 21.59	19.65 ± 20.86	18.38 ± 23.27	0.66
Corneal sensation (value from right eye) (mean ± SD)				
Belmonte Esthesiometer (mL/min, range 0–200)	84.04 ± 39.71	83.49 ± 40.92	102.55 ± 67.10	**0.045**
Dry eye signs (value from more severely affected eye) (mean ± SD)				
InflammaDry (range 0–3)	0.60 ± 0.72	0.70 ± 0.80	0.85 ± 0.99	0.23
Upper eyelid laxity (range 0–2)	0.59 ± 0.68	0.67 ± 0.69	0.71 ± 0.75	0.55
Lower eyelid laxity (range 0–2)	0.52 ± 0.60	0.46 ± 0.62	0.56 ± 0.67	0.58
Anterior blepharitis (range 0–3)	0.49 ± 0.60	0.51 ± 0.63	0.68 ± 0.78	0.46
Eyelid vascularity (range 0–3)	0.55± 0.78	0.57± 0.72	0.57 ± 0.80	0.98
MG plugging (range 0–3)	1.74 ± 0.90	1.79 ± 0.96	1.52 ± 0.67	0.25
MG dropout (range 0–4)	1.47 ± 0.95	1.69 ± 1.16	1.69 ± 1.20	0.20
Papillae (range 0–3)	0.61 ± 0.71	0.55 ± 0.67	0.64 ± 0.85	0.81
Conjunctivochalasis (range 0–3)	0.50 ± 0.33	0.50 ± 0.33	0.54 ± 0.34	0.80
TBUT, seconds	9.04 ± 4.15	8.78 ± 4.64	9.41 ± 4.78	0.70
Corneal staining (range 0–15)	1.86 ± 2.22	2.10 ± 2.59	2.38 ± 2.85	0.44
Schirmer, mm wetting at 5 min	13.11 ± 7.46	12.46 ± 7.07	14.57 ± 7.88	0.26
Meibum quality (range 0–4)	1.88 ± 1.35	2.08 ± 1.30	1.95 ± 1.29	0.42

DEQ-5: Dry Eye Questionnaire-5; OSDI: Ocular Surface Disease Index; NPSI: Neuropathic Pain Symptoms Inventory; TBUT: tear break up time; MG: Meibomian glands; OD: right eye.

**Table 3:** Anti-inflammatory therapy for each genotype.

Anti-Inflammatory Therapy	Genotypes			Total
	TT (*n* = 16)	CT (*n* = 10)	CC (*n* = 4)	
Cyclosporine 0.05%	43.3% (13)	26.7% (8)	10.0% (3)	80.0% (24)
Lifitegrast 5%	10.0% (3)	6.7% (2)	3.3% (1)	20.0% (6)

## Data Availability

Data available upon reasonable request.
